# Corticolimbic Functional Connectivity in Adolescents with Bipolar Disorder

**DOI:** 10.1371/journal.pone.0050177

**Published:** 2012-11-21

**Authors:** Fei Wang, Laurel Bobrow, Jie Liu, Linda Spencer, Hilary P. Blumberg

**Affiliations:** 1 Department of Psychiatry, Yale School of Medicine, New Haven, Connecticut, United States of America; 2 Child Study Center, Yale School of Medicine, New Haven, Connecticut, United States of America; 3 Department of Diagnostic Radiology, Yale School of Medicine, New Haven, Connecticut, United States of America; The University of Melbourne, Australia

## Abstract

Convergent evidence supports regional dysfunction within a corticolimbic neural system that subserves emotional processing and regulation in adolescents and adults with bipolar disorder (BD), with abnormalities prominent within the amygdala and its major anterior paralimbic cortical connection sites including ventral anterior cingulate, orbitofrontal, insular and temporopolar cortices. Recent studies of adults with BD demonstrate abnormalities in the functional connectivity between the amygdala and anterior paralimbic regions suggesting an important role for the connections between these regions in the development of the disorder. This study tests the hypothesis that these functional connectivity abnormalities are present in adolescents with BD. Fifty-seven adolescents, twenty-one with BD and thirty-six healthy comparison (HC) adolescents, participated in functional magnetic resonance imaging while processing emotional face stimuli. The BD and HC groups were compared in the strength of functional connectivity from amygdala to the anterior paralimbic cortical regions, and explored in remaining brain regions. Functional connectivity was decreased in the BD group, compared to the HC group, during processing of emotional faces in ventral anterior cingulate (VACC), orbitofrontal, insular and temporopolar cortices (p<0.005). Orbitofrontal and VACC findings for the happy condition, and additionally right insula for the neutral condition, survived multiple comparison correction. Exploratory analyses did not reveal additional regions of group differences. This study provides evidence for decreased functional connectivity between the amygdala and anterior paralimbic cortices in adolescents with BD. This suggests that amygdala-anterior paralimbic connectivity abnormalities are early features of BD that emerge at least by adolescence in the disorder.

## Introduction

Dysfunction in the corticolimbic neural system that subserves emotional processing and regulation is a consistent finding in bipolar disorder (BD) [1]. The amygdala is a core component in this system and amygdala abnormalities are amongst the most consistent findings in adolescents with BD [2], [3], [4], [5], [6], [7], [8]. The anterior paralimbic cortices are the cortical regions with some of the strongest connectivity to the amygdala [9], [10], and include ventral anterior cingulate cortex (VACC), orbitofrontal cortex (OFC), insular cortex (IC) and temporopolar cortex (TPC). Together with the amygdala, the anterior paralimbic structures comprise critical elements in this system and in the emotional functions disrupted in BD.

Regional abnormalities within the amygdala and anterior paralimbic structures have been observed for several decades in adults with BD and studies are increasingly demonstrating abnormalities in adolescents with BD. Both structural abnormalities, as well as functional abnormalities in response to face stimuli, within the amygdala have been consistent findings in adolescents with BD [2], [3], [4], [5], [6], [7], [8]. While there are fewer reports of abnormalities within anterior paralimbic structures in adolescents with BD, these are increasingly emerging. Structural neuroimaging studies of adolescents with BD report abnormalities within one or more anterior paralimbic region including the ACC, OFC and TPC [11], [12], [13], [14], [15], [16], [17]. We recently observed volume decreases within OFC, IC and TPC in adolescents with BD, compared to healthy adolescents [18], suggesting that abnormalities in these anterior paralimbic structures may develop in concert and become central cortical abnormalities in adolescents with the disorder. Dysregulated responses within OFC and IC have been reported in functional neuroimaging studies of adolescents with BD during emotional processing [7], [8], [19], [20], [21], [22].

Increasing evidence implicates abnormalities in the connections between the amygdala and anterior paralimbic cortices in adolescents with BD. The conventional functional magnetic resonance imaging (fMRI) activation studies above provided information about the functioning within these brain regions. Functional connectivity measures the coupling of activity between regions in time [23]. Corticolimbic functional connectivity abnormalities between amygdala and VACC or OFC in adults with BD have been observed in individuals at rest and during performance of emotional face processing tasks [24], [25], [26], [27], [28], [29], [30], suggesting functional connectivity alterations may be an important feature of the disorder.

The regional abnormalities in the amygdala and paralimbic cortices reported in adolescents suggest the functional connectivity abnormalities may be present in adolescents with the disorder as well; however, such abnormalities have not been detected previously in studies of children and adolescents with BD. One functional connectivity study, in youths ages 7 to 17 years at rest, did not show connectivity abnormalities to an amygdala seed region, although connectivity abnormalities were detected to the superior temporal gyrus [31]. Another study of youths ages 7 to 18 years with BD during the processing of face stimuli did show reduced functional connectivity from amygdala although this was detected to posterior association cortices [32].

In this fMRI study, adolescents performed an emotional face processing task that included faces depicting fearful and neutral expressions as well as positive expressions, as the latter have been suggested to be especially helpful in eliciting BD-related corticolimbic pathology [7], [25], [33], [34], [35], [36], [37]. Bilateral amygdala was used as the seed region for functional connectivity analyses as amygdala findings appear to be consistent findings during adolescence in BD, as described above. We tested the hypothesis that functional connectivity is reduced from amygdala to anterior paralimbic cortices in adolescents with BD, compared to HC adolescents. Whole brain analyses were performed to explore for other potential regions of functional connectivity differences.

## Methods

### Subjects

The HC group was comprised of 36 adolescents [ages 13–18 years, mean age  = 15.75±1.90 years, 19 (53%) female]. The BD group was comprised of 21 adolescents who met DSM-IV diagnosis for BD-I [ages 13–18 years, mean age  = 15.81±1.81 years, 10 (48%) female]. Raw fMRI data from some of the subjects of this study were used in previous studies of regional brain activation, including 11 each of the BD and HC participants [35], 2 HC participants [38] and 1 BD and 1 HC participant [39]; functional connectivity analyses were not performed in those studies. Participants were without a history of other neurological disorder or loss of consciousness longer than five minutes, significant medical disorders or alcohol or other substance dependence/abuse except one participant with BD with cannabis dependence in remission 4 months. The presence or absence of DSM-IV Axis I disorders and mood state at scanning were confirmed by administration of the revised Schedule for Affective Disorders and Schizophrenia – Present and Lifetime Version (KSAD-PL) to participants under 18 years of age and their parents/guardians, or the Structural Clinical Interview for DSM-IV Axis-I Disorders (SCID) for participants aged 18 years. Final DSM-IV diagnoses were established by the consensus of the structured interviews and clinical interviews by board-certified psychiatrists and psychologists expert in childhood mood disorders. At the time of scanning, 6 (28.6%) BD participants met criteria for an elevated mood episode (manic, mixed, or hypomanic), 4 (19.0%) for a depressive episode, and 11 (52.4%) were euthymic. The severity of manic symptoms was determined by the Young Mania Rating Scale (YMRS) (mean±standard deviation: 4.57±5.72). The severity of depressive symptoms was determined by the Children's Depression Rating Scale (CDRS) (mean±standard deviation: 26.10±9.25). Seven (33.3%) participants with BD met criteria for rapid-cycling, 4 (19.0%) reported chronic daily cycling in addition to having experienced a well-defined elevated mood episode of duration consistent with DSM-IV criteria. Three (14.3%) of the BD participants were unmedicated at the time of scanning. Psychotropic medications prescribed to the remaining participants with BD at the time of scanning included lithium carbonate (5, 23.8%), anticonvulsants (9, 42.9%), atypical antipsychotics (12, 57.1%), antidepressants (6, 28.6%) and stimulants (8, 38.1%). Two of the subjects were prescribed only one medication. Comorbid psychiatric illnesses included 6 BD participants with attention-deficit/hyperactivity disorder (ADHD), one with obsessive-compulsive disorder, one with specific phobia and one with panic disorder.

After complete description of the study, written informed consent was obtained from parents/guardians and participants 18 years of age. Written informed assent was obtained from minors. This research was approved by the Yale University Institutional Review Board.

#### MRI Data Acquisition

FMRI data were acquired with a 3-Tesla Siemens Trio MR scanner (Siemens, Erlangen, Germany) with a single-shot echo planar imaging sequence in alignment with the anterior commissure-posterior commissure plane with the parameters: TR = 2000 ms; TE = 25 ms; matrix = 64×64; FOV = 240×240 mm^2^ and 32 three-mm slices without gap.

#### Emotional Face Paradigm

During fMRI scanning, an event-related emotional face task was completed by each participant. Participants viewed faces from the Ekman series depicting fearful, happy or neutral expressions and were instructed to press a button to make a male-female determination, as described previously [24], [33], [35]. In brief, 5 male and 5 female faces were each presented for 2 seconds with interstimulus intervals of 4, 8 or 12 seconds. Each of the three expressions was shown for each actor, for a total of 30 face stimuli in each 4 minute, 50 second run. There were 4 runs, and ordering of face stimuli were varied systematically to control for sequential dependencies. Runs were counterbalanced for the identity, sex and expression of the faces, as well as for the interstimulus intervals.

#### Functional Connectivity Processing

Statistical Parametric Mapping 8 (SPM8) software (http://www.fil.ion.ucl.ac.uk/spm) was used for BOLD fMRI pre-processing. For each run, the initial two images were discarded, the remaining images were corrected for within-scan acquisition time differences between slices and realigned to the first volume to correct for inter-scan movements. Linear motion (x, y, z planes) for all participants was below 2.5 mm and rotational motion (pitch, roll, yaw) below 2.5 degrees. FMRI data were then spatially normalized to Montreal Neurological Institute space, resampled to 3×3×3 mm^3^ and spatially smoothed (8 mm full width at half maximum). Low-frequency noise was removed via a high pass filter (128 s).

The bilateral amygdala seed region of interest (ROI) was defined with the WFU Pick Atlas Tool (http://www.fmri.wfubmc.edu/download.htm). For each subject, a mean time series for the amygdala seed ROI was calculated by averaging the time series for all voxels within the amygdala ROI separately for each face emotion type (fearful, happy, neutral). Correlational analyses were then performed between the amygdala time series and the time series for each brain voxel [24], resulting in a correlation map for each subject that contained the correlation coefficient for each voxel with that of the amygdala ROI for each face emotion type. The correlation coefficients for each face emotion type were transformed to Z values using Fisher r-to-z transformation for further statistical testing.

**Figure 1:**
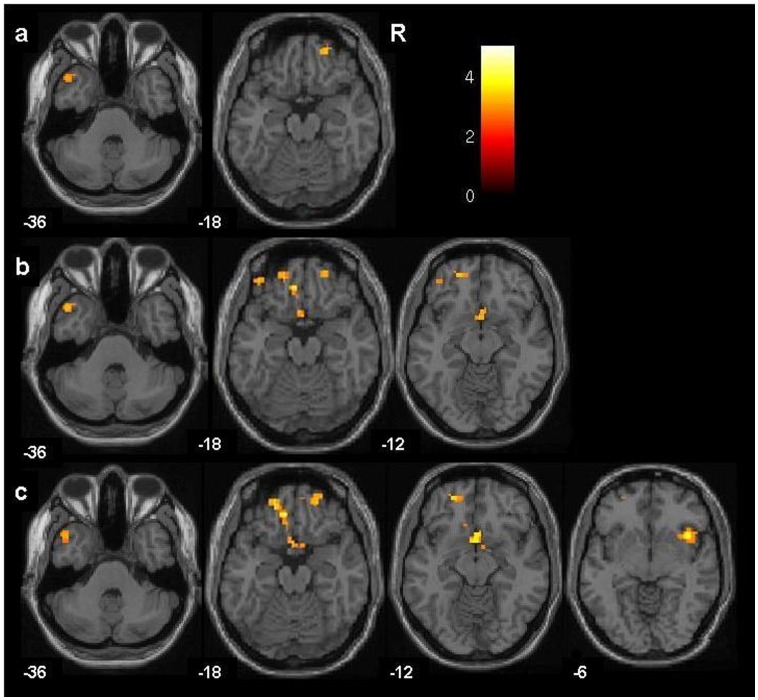
The axial-oblique images display regions where functional connectivity was significantly decreased (p<0.005, cluster>20) in 21 adolescents with bipolar disorder, compared to 36 healthy comparison participants during fearful (a), happy (b) and neutral (c) faces processing. The color bar represents the range of T values. Montreal Neurological Institute z-plane coordinates (millimeters) are presented. R = right.

#### Statistical Analyses

Group differences were explored throughout the whole brain using voxel-based analysis of covariance (ANCOVA), including age and sex as covariates, with the functional connectivity correlation coefficients (Z scores) from the amygdala to all brain voxels as the dependent variables for each face condition (fearful, happy and neutral face expressions). Consistent with previous studies [24], [40], findings in hypothesized anterior paralimbic regions were considered significant at *P*<0.005 and spatial extent of 20 voxels. Analyses were also performed with small volume correction (SVC) for multiple comparisons [*P*<0.05, corrected with false discovery rate (FDR)] to further confirm the findings for the anterior paralimbic regions. For remaining brain regions, findings were considered as significant with *P*<0.05 corrected for FDR and an extent threshold of 20 voxels. For anterior paralimbic regions showing significant differences between the HC and BD groups, post-hoc exploratory ANCOVA analyses were performed analogous to those above, but including data only for BD participants, for potential main effects of mood state (euthymic, depressed and manic), rapid-cycling (yes/chronic/no), comorbidity of ADHD (yes/no) or medication status [(overall (yes/no), lithium carbonate (yes/no), anticonvulsants (yes/no), atypical antipsychotics (yes/no), antidepressants (yes/no) and stimulants (yes/no)] on these regions by assessing each of these factors one at a time in the model. Additionally, potential associations between functional connectivity of anterior paralimbic regions showing significant differences between the HC and BD groups, and YMRS/CDRS among BD participants were examined using correlation analysis. Findings in these analyses were considered significant at *P*<0.005 and spatial extent of 20 voxels.

## Results

The HC and BD groups did not differ significantly in age (*P* = 0.98) or sex (*P* = 0.61).

For the anterior paralimbic structures, in adolescents with BD compared to HC adolescents, functional connectivity was significantly decreased from the amygdala to the left TPC for the three face conditions, to bilateral OFC and VACC for happy and neutral face conditions, to right OFC for the fear face condition, and to right IC for the neutral face condition (Figure 1, Table 1). The findings of bilateral OFC and VACC for happy and neutral face conditions, and right insula for the neutral condition, remained significant with SVC. Exploratory analyses did not reveal significant differences in remaining brain regions. Post-hoc exploratory analyses performed in BD participants did not reveal any significant effects of mood state, YMRS or CDRS scores, rapid-cycling, medication status, or ADHD comorbidity on functional connectivity to anterior paralimbic cortices.

**Table 1:** Areas of reduced functional connectivity during emotional face processing in adolescents with bipolar disorder compared to health control adolescents.

		MNI coordinates			
Cortical Regions (Brodmann Areas)	Cluster Size	x	y	z	T values
**Fearful Face**					
Right Orbitofrontal (11)	20	24	54	−18	3.31
Left Temporal Pole (38)	23	−45	15	−39	3.00
**Happy Face**					
Left Orbitofrontal (11/47)*	62	−9	39	−21	3.77
Right Orbitofrontal (11)*	37	21	54	−21	3.45
Bilateral Ventral Anterior Cingulate Cortex (25)*	20	0	12	−15	3.56
Left Temporal Pole (38)	20	−45	15	−36	3.19
**Neutral Face**					
Left Orbitofrontal (11/47)*	191	−18	48	−18	4.12
Right Orbitofrontal (11)*	66	21	54	−21	3.46
Bilateral Ventral Anterior Cingulate Cortex (25)*	38	0	9	−12	3.96
Right Insular Cortex*	70	39	15	−6	3.95
Left Temporal Pole (38)	25	−45	12	−39	3.20

* survived small volume correction.

MNI: Montreal Neurological Institute.

## Discussion

We found decreased functional connectivity from the amygdala to anterior paralimbic cortices in adolescents with BD, compared to the HC adolescents, during processing of emotional faces. Specifically, decreased functional connectivity in the BD group was observed in the VACC, OFC, IC and TPC. The OFC and VACC findings for the happy condition, and additionally right insula findings for the neutral condition, survived multiple comparison correction.

The current study identified altered functional connectivity from the amygdala to the anterior paralimbic cortices in adolescents with BD during processing of emotional faces. Adolescence is a critical period in the development of connections from the amygdala to the anterior paralimbic cortices thought to contribute to the maturation of emotional and social responses [41]. Interestingly, a recent study supports our findings of reduced ventral prefrontal cortex-amygdala connectivity to fearful faces in BD youth [42]. Anterior paralimbic cortices are a group of highly interconnected structures that are thought to develop synchronously to comprise critical elements in these behaviors [10]. Taken together with strongly convergent previous findings in amygdala [2], [3], [4], [5], [6], [7], [8], as well as our recent report of reduced volumes in anterior paralimbic structures [18], in adolescents with BD, the new findings suggest that amygdala-anterior paralimbic neural circuitry may play an important role in the development of BD and its presentation in adolescents. Despite the important role amygdala-paralimbic circuitry is thought to play in adolescence, and its involvement in psychiatric disorders, the development of this circuitry in adolescence has received little direct study in animal and human models. Further preclinical and clinical studies of this circuitry are critically needed and may yield important new perspectives on the development of BD.

The findings reported herein are consistent with findings reported previously in adults with BD, extending the findings to adolescents with BD and potentially implicating additional regions of impaired connectivity. We previously demonstrated a disruption in the functional connections between the VACC and amygdala during emotional processing in adults with BD (mean age: 32 years) [24]. In that study, we used a VACC seed region that limited ability to assess the functional connections from the amygdala to other anterior paralimbic regions. Three studies of adults with BD (mean ages: 33–43 years), at rest or while processing emotional faces, have shown decreased functional connectivity between the amygdala and OFC [25], [26], [27]. A recent effective connectivity analysis found distinct patterns of functional connectivity between the amygdala and dorsomedial and ventrolateral prefrontal cortex in remitted BD and depressed BD participants [29]. We are not aware of previous reports of findings of decreased functional connectivity with TPC in BD. TPC activation abnormalities have been observed in adults with BD during emotional face processing [43]. Insula activation abnormalities have been observed in both adults and adolescents with BD [7], [21], [44]. Altered functional connectivity between the amygdala and IC was also recently identified in depressed BD adults [30]. Given the roles of the TPC and IC in the integration of extrapersonal sensory and internal state information, and in the processing of the emotional states of others [44], [45], this suggests roles for the connections between the amygdala and these structures in emotional and social difficulties experienced by individuals with BD. We speculate that the insula findings observed only during neutral face processing might relate to differences in the integration of internal state information while processing the emotionally ambiguous neutral stimuli.

This study suggests that these functional connectivity abnormalities may be features of the disorder present by at least adolescence. However, it cannot be determined from this study whether the connectivity abnormalities are present prior to adolescence and what the developmental trajectory of the abnormalities are. Future longitudinal studies of pre-adolescents and adolescents with BD, or at high risk for BD, are needed.

We did not detect significant main effects of clinical variables, such as the presence or absence of rapid-cycling, mood states or symptoms, medication status or ADHD comorbidity within the BD group on the functional connectivity between the amygdala and anterior paralimbic cortices. However, power to detect differences may have been limited. Factors such as medication were not varied systematically and the majority of BD participants were prescribed more than one medication. Although we are not aware of previous findings of medication effects on this functional connectivity in BD, future studies with unmedicated participants assigned systematically to treatments would provide more conclusive evidence.

In summary, this study provides evidence for decreased functional connectivity between the amygdala and anterior paralimbic cortices in adolescents with BD. This suggests amygdala-anterior paralimbic connectivity abnormalities may be early abnormalities in BD that emerge at least by adolescence in the disorder.
